# Evaluation of a continuous blood glucose monitoring system using central venous microdialysis

**DOI:** 10.1186/cc9825

**Published:** 2011-03-11

**Authors:** F Möller, J Liska, A Franco-Cereceda

**Affiliations:** 1 Karolinska Institutet, Stockholm, Sweden

## Introduction

Glycemic control in critically ill patients has been shown to be beneficial. In this prospective study we therefore evaluated the accuracy and technical feasibility of a continuous glucose monitoring system using intravascular microdialysis.

## Methods

Fifty patients undergoing cardiac surgery were monitored using a 4Fr intravenous microdialysis catheter (Eirus SLC^®^; CMA Micro-dialysis AB, Solna, Sweden), percutaneously placed with the tip of the catheter positioned in the superior vena cava. The catheter was connected to the Eirus monitoring system and the patients were monitored for up to 48 hours postoperatively in the ICU. As reference, arterial blood samples were taken every hour and analyzed in a blood gas analyzer (ABL800 FLEX^®^; Radiometer Medical, Copenhagen, Denmark).

## Results

**Figure 1:**
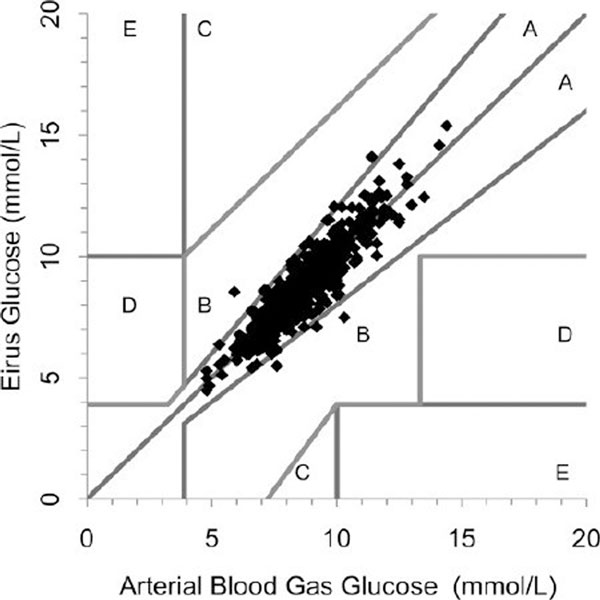
**Error grid analysis of arterial blood gas and microdialysis glucose samples**.

Data were available from 48 patients. A total of 994 paired (arterial blood gas-microdialysis) samples were obtained. The glucose correlation coefficient (*R*^2^) was 0.85. Using Clarke error grid analysis, 100% of the paired samples were in region AB and 99% in region A Figure 1). The mean glucose level was 8.3 mmol/l, bias 0.2% and the mean absolute relative difference was 5%. A total 99.2% of the paired samples were correct according to ISO criteria. Bland-Altman analysis showed bias ± limits of agreement were 0.02 ± 1.1 mmol/l.

## Conclusions

Central venous microdialysis is a highly accurate and reliable method for continuous blood glucose monitoring up to 48 hours in ICU patients undergoing cardiac surgery. The system may thus be useful in critically ill ICU patients.

